# Drowning prevention knowledge and risk behaviors among children and adolescents in China: a cross-sectional study

**DOI:** 10.3389/fpubh.2026.1821083

**Published:** 2026-04-14

**Authors:** Zhaohao Zhong, Sijia Wang, Yinchao Zhu, Jinyu Xue, Wangwei Lou, Tao Zhang, Jieping Chen

**Affiliations:** 1 Ningbo Municipal Centre for Disease Control and Prevention, Ningbo, China; 2 Xiangshan County Center for Disease Control and Prevention, Ningbo, China; 3 Ninghai County Center for Disease Control and Prevention, Ningbo, China

**Keywords:** awareness, children and adolescents, demographic factors, drowning prevention, risky behaviors

## Abstract

**Background:**

Drowning is a leading cause of death among children and adolescents worldwide, with China being one of the most affected countries. Despite relatively high levels of drowning prevention knowledge, many children and adolescents continue to engage in risky water-related behaviors. This study aimed to assess drowning prevention knowledge, the prevalence of risky behaviors, and demographic factors associated with these outcomes among children and adolescents in Ningbo, China.

**Methods:**

A cross-sectional survey was conducted with 4,759 adolescents from schools across Ningbo, China. Data were collected using a structured questionnaire that assessed sociodemographic characteristics, drowning prevention knowledge, and risky behaviors related to water activities. Statistical analyses, including chi-square tests and multivariate logistic regression, were used to identify factors influencing drowning knowledge and behavior.

**Results:**

The study found that while most participants (over 90%) demonstrated awareness of basic drowning prevention principles, such as the importance of adult supervision and not swimming alone, a significant proportion still engaged in risky behaviors. Males, older adolescents, students with non-local household registration, and boarding students exhibited significantly lower levels of drowning prevention knowledge. Multivariate logistic regression analysis indicated that males were more likely to engage in drowning-related risky behaviors, whereas mastery of drowning prevention knowledge served as a protective factor against such behaviors.

**Conclusion:**

Although awareness of drowning prevention is high, risky behaviors persist, particularly among high-risk groups such as males, students with non-local household registration. Targeted interventions that combine knowledge enhancement with behavioral and social strategies are needed to bridge the gap between awareness and safe practices. Future programs should prioritize practical safety education, swimming skills training, and community support to reduce drowning risk among vulnerable youth populations.

## Introduction

Due to the acceleration of urbanization, the rapid development of industry and infrastructure, and significant changes in living environments, risk factors for children have increased, making the issue of child injuries increasingly prominent (1). As the world’s largest developing country, injuries are the leading cause of death for children aged 1–15 in China and a major contributor to disabilities among adolescence (2). As one of the major causes of injury-related incidents, drowning is the second leading cause of death among children and adolescents (3). The World Health Organization (WHO) defines drowning as the process of experiencing respiratory impairment from submersion or immersion in liquid (4). According to the World Health Organization’s 2024 report, approximately 300,000 people worldwide die from drowning each year, children and young people bear the largest burden (5). Those under 5 bear the highest burden, accounting for 24% of all drowning deaths, followed by children aged 5–14 at 19%, and young people aged 15–29 at 14%, and low- and middle-income countries remain worst affected (5). Nowadays, drowning has become a major public health problem worldwide and not only causes irreparable physical and psychological trauma to children but also places a heavy burden on society and families, resulting in significant economic losses.

Previous studies have indicated that there are numerous risk factors for drowning among children and adolescents, including young age, male gender, summer season, alcohol intoxication, and insufficient swimming ability (6–8). And it is estimated that greater than 90% of drownings are preventable (9). Currently, numerous interventions targeting children and adolescents have been implemented, including but not limited to swimming skill training, environmental modifications, safety supporting educational programs, day-care services for pre-school children, etc. (10, 11). In China, some scholars have successfully applied an interactive group-based educational intervention to improve minors’ knowledge of drowning prevention, as well as the prevention of other unintentional injuries such as falls (12). And it was proven to be effective and worth popularizing. Overall, current interventions aimed at preventing drowning among children and adolescents are mainly based on the “5E” principle, and the comprehensive “5E” intervention has increasingly gained consensus among experts in the field of unintentional injury prevention in children (13, 14). The “5E” framework primarily includes educational intervention, engineering intervention, enforcement intervention, economic intervention, and emergency care and first aid.

The root cause of drowning incidents frequently lies in unsafe behaviors driven by a lack of awareness (15). Adolescents, with their strong curiosity and insufficient risk perception, tend to underestimate water-related dangers such as undercurrents, sudden temperature changes, and abrupt depth variations. Studies also indicate that drownings among adolescents mostly occur in unsupervised natural water bodies or during brief parental absence (16). Awareness education can address such supervision gaps, encouraging young people to become the “first responders” for their own safety. Proactive prevention awareness acts as an “invisible safety net” that functions anytime and anywhere, with low costs and broad coverage, fundamentally reducing risky behaviors. Adolescents equipped with drowning prevention awareness not only protect themselves but may also influence their peers—for instance, by discouraging unsafe swimming or calling for help in emergencies. This ripple effect can foster a culture of safety, collectively reducing drowning incidents from the individual to the community level. Drowning prevention awareness represents the most cost-effective, sustainable, and far-reaching intervention. It changes behavioral patterns at the source, empowers adolescents to actively avoid dangers, and extends its impact to families and society at large. While measures such as swimming skills training, environmental improvements, and enhanced supervision remain crucial, their effectiveness is significantly diminished without a foundation of awareness. Therefore, within a multi-layered drowning prevention framework, raising awareness serves as the core pillar and should be prioritized as the primary focus of intervention efforts.

Ningbo, a coastal city in Zhejiang Province in eastern China, is characterized by numerous water bodies and a subtropical monsoon climate with hot and rainy summers and autumns. Drowning has become a significant public health concern in this region (17). Given this, we conducted this study to investigate the knowledge and behaviors related to drowning among different groups of children and adolescents in Ningbo. By performing a comparative analysis across various groups (such as gender, age, and whether they are left-behind children), the study aims to inform the development of targeted educational interventions. Through this comparative analysis, we seek to understand the differences in their knowledge and behaviors regarding drowning, in order to design more tailored interventions in the future to enhance their drowning prevention awareness.

## Methods

### Participants

Upon completion of the questionnaire design and a pilot survey, a formal investigation was conducted in selected primary schools and middle schools. The sample size was evenly distributed across two districts/counties in Ningbo, and primary and secondary schools within these areas were invited to participate in the survey using simple random sampling. The research team consisted of investigators from local health centers and in-school teachers, all of whom received uniform training prior to the formal survey. The questionnaire was completed by students through self-administration in a centralized setting organized by the schools. The project staff distributed the questionnaires to the schools, where designated personnel were responsible for gathering the selected students. With the assistance of school teachers, the project staff provided unified instructions on how to complete the questionnaire. The students completed the questionnaires on site, and the completed questionnaires were collected immediately by the staff. To ensure survey quality and minimize bias, each survey was conducted with the support of school administrators and the cooperation of homeroom teachers. Before filling out the questionnaire, investigators explained the precautions to the students and promptly answered their questions. Meanwhile, informed consent was obtained from all adolescents and their parents prior to the formal start of the survey.

### Inclusion criteria and exclusion criteria

The inclusion criteria were (1) Children and adolescents in Grade 4 and above (to avoid potential difficulties in independently completing the questionnaire due to younger age and to ensure the accuracy and completeness of the responses). (2) voluntarily participate in the survey, (3) able to complete the questionnaire independently. (4) no somatic diseases, (5) no psychiatric diseases or abnormalities currently.

The exclusion criteria were (1) decline to participate in survey or refuse to provide informed consent, (2) clinically diagnosed somatic or psychiatric diseases and abnormalities.

### Sample size

The formula used to calculate the sample size for the baseline survey is:


$$N=\frac{z_{\alpha }^{2}\times p(1-p)}{d^{2}}$$

In the formula, *p* represents the prevalence of swimming in unsafe places, which was set at 11.8% (18). *z* is the critical value of the standard normal distribution, and *α* represents the significance level. When *α* = 0.05, *z_α_* = 1.96. *d* is the allowable error, defined as d = 0.1 × *P*. Considering potential non-responses and invalid questionnaires, the calculated sample size was increased by 20%. Therefore, the minimum required sample size for this baseline survey was 3,446 participants. After excluding invalid responses, 4,759 valid questionnaires were retained for analysis.

### Measurement

We adopted the section on “Drowning Prevention Survey” from the “2016-2020 Child Injury Prevention Project – Thematic Survey on Child Injury” developed by the Chinese Center for Disease Control and Prevention. The questionnaire covered sociodemographic information, including gender, age, grade, household registration status, boarding status, and left-behind status. Drowning prevention knowledge included eight items, these questions covered several aspects of water safety, including general awareness that drowning is preventable, safe swimming practices (such as avoiding swimming alone and performing warm-up exercises before swimming), the importance of adult supervision and avoiding unsafe water environments, and appropriate responses during drowning emergencies, particularly safe rescue practices. Participants who answered all eight questions correctly were considered to have mastered drowning prevention knowledge. Regarding drowning-related risky behaviors, two items were included: “Water activities at unsupervised locations with no lifeguard/equipment” and “Water activities accompanied by a guardian with no lifeguard/equipment.”

### Statistical analysis

The collected questionnaires were reviewed and verified, followed by double data entry using Epidata 3.1 to ensure quality, and subsequent analysis was performed with R 4.2.1. Categorical variables were described using frequency and percentage, and continuous variables were described using means and standard deviation (SD) with normal distribution and median (range) without normal distribution. Categorical data were compared using the chi-square test. Based on the results of univariate analysis, variables were selected for inclusion in a multivariate logistic regression model. The multivariate analysis employed a forward stepwise regression method, whereby variables with *p* < 0.05 were included in the multivariate model and those with *p* > 0.1 were removed. This process identified influencing factors for drowning-related risk behaviors among participans, which were then described using odds ratios (*OR*) with 95% confidence intervals (95% *CI*).

## Results

### Characteristics of participants

The characteristics of 4,759 adolescents participated in the survey as shown in Table 1. The surveyed adolescents had an average age of 14.54 years (range: 2.62 years). Among them, there were 2,348 (49.34%) males and 2,411 (50.66%) females. Boarding students accounted for 65.43% (1,686/4759), while left-behind children accounted for 5.59% (266/4759). Over the past year, 46.31% (2,204/4759) of the adolescents had engaged in aquatic activities. Meanwhile, the survey found that only 15.53% (739/4759) of the adolescents clearly indicated that they were capable of swimming continuously for 25 meters and treading water for more than 30 s.

**Table 1:** Basic characteristics of the participants, *n* (%).

Variables	Categories	Total (*n* = 4,759)
Sex		
	Males	2,348 (49.34)
	Females	2,411 (50.66)
Grade		
	High School	1723 (36.21)
	Junior school	1,521 (31.96)
	Primary school	1,515 (31.83)
Household registration		
	Local	3,916 (82.29)
	Non-local	843 (17.71)
Boarding status		
	Yes	1,686 (35.43)
	No	3,073 (64.57)
Left-behind children		
	Yes	266 (5.59)
	No	4,493 (94.41)
Capable of swimming 25 m and treading water >30s		
	Yes	739 (15.53)
	No	2,984 (62.70)
	Not clear	1,036 (21.77)
Frequency of aquatic activities (e.g., swimming, boating) in the Past Year		
	None	2,555 (53.69)
	1–4 times	1,252 (26.31)
	5–9 times	334 (7.01)
	10 times and above	618 (12.99)

### Knowledge of drowning prevention

The drowning prevention knowledge among the surveyed adolescents is shown in Table 2. The item with the lowest awareness rate was “Don’t attempt to form a human chain to rescue a drowning person,” at only 77.56% (3,691/4759). The second lowest was “Even skilled swimmers should never jump in immediately to save a drowning person,” at 88.91% (4,231/4759). The awareness rates for all other drowning prevention knowledge items exceeded 90.00%. Among them, males showed significantly lower mastery of drowning prevention knowledge than females. Regarding grade level, except for the item “Warm-up exercises should be performed prior to swimming”, significant differences were observed across grades in the mastery of all other knowledge items. Primary school students ranked first in six out of the eight drowning knowledge items. Adolescents with local household registration demonstrated higher mastery of drowning prevention knowledge than those with non-local household registration, and day students scored higher than boarding students. Although left-behind children demonstrated lower knowledge of drowning compared to non-left-behind children, the difference did not reach statistical significance.

**Table 2:** Comparison of drowning knowledge mastery across different groups, *n* (%).

Knowledge of drowning prevention	Awareness status	Gender				Grade			Household registration				Boarding status left-behind children									
		Male	Female	*χ* ^2^	*P*	Primary school	Junior school	High school	*χ* ^2^	*P*	Local	Non-local	*χ* ^2^	*P*	Yes	No	*χ* ^2^	*P*	Yes	No	*χ* ^2^	*P*
Drowning is preventable	4,402 (92.50)	2,159 (91.95)	2,243 (93.03)	5.976	**0.050**	1,385 (91.42)	1,420 (93.63)	1,597 (92.69)	48.742	**<0.001**	3,653 (93.28)	749 (88.85)	48.742	**<0.001**	1,555 (92.23)	2,847 (92.65)	1.021	0.600	243 (91.35)	4,159 (92.57)	0.648	0.723
You should not swim alone, even if you are a strong swimmer	4,669 (98.11)	2,284 (97.27)	2,385 (98.92)	21.844	**<0.001**	1,495 (98.68)	1,493 (98.16)	1,681 (97.56)	13.505	**0.009**	3,851 (98.34)	818 (97.03)	19.257	**<0.001**	1,643 (97.45)	3,026 (98.47)	10.051	**0.007**	260 (97.74)	4,409 (98.13)	0.202	0.904
Children should always have adult supervision when swimming, even with peers.	4,615 (96.97)	2,252 (95.91)	2,363 (98.01)	19.075	**<0.001**	1,496 (98.75)	1,476 (97.04)	1,643 (95.36)	39.752	**<0.001**	3,799 (97.01)	816 (96.80)	5.119	0.077	1,608 (95.37)	3,007 (97.85)	30.734	**<0.001**	256 (96.24)	4,359 (97.02)	1.200	0.549
Swimming and water play are prohibited at unsupervised locations without lifeguards or safety equipment, even with adult supervision.	4,410 (92.67)	2,136 (90.97)	2,274 (94.32)	32.060	**<0.001**	1,432 (94.52)	1,390 (91.39)	1,588 (92.16)	18.999	**0.001**	3,644 (93.05)	766 (90.87)	8.861	**0.012**	1,551 (91.99)	2,859 (93.04)	9.629	**0.008**	241 (90.60)	4,169 (92.79)	1.801	0.406
When a companion is drowning, you should never enter the water to attempt a rescue by yourself.	4,426 (93.00)	2,138 (91.06)	2,288 (94.90)	31.198	**<0.001**	1,469 (96.95)	1,423 (93.56)	1,534 (89.03)	79.979	**<0.001**	3,643 (93.03)	783 (92.88)	0.257	0.879	1,497 (88.79)	2,929 (95.31)	72.956	**<0.001**	243 (91.35)	4,183 (93.10)	2.683	0.261
Warm-up exercises should be performed prior to swimming.	4,638 (97.46)	2,266 (96.51)	2,372 (98.38)	18.315	**<0.001**	1,472 (97.16)	1,487 (97.76)	1,679 (97.45)	3.272	0.513	3,826 (97.70)	812 (96.32)	8.931	**0.011**	1,642 (97.39)	2,996 (97.49)	0.096	0.953	257 (96.62)	4,381 (97.51)	1.477	0.478
Do not attempt to form a human chain to rescue a drowning person.	3,691 (77.56)	1748 (74.45)	1943 (80.59)	28.247	**<0.001**	1,399 (92.34)	1,128 (74.16)	1,164 (67.56)	307.808	**<0.001**	3,002 (76.66)	689 (81.73)	10.604	**0.005**	1,150 (68.21)	2,541 (82.69)	134.261	**<0.001**	196 (73.68)	3,495 (77.79)	2.442	0.295
Even skilled swimmers should never jump in immediately to save a drowning person.	4,231 (88.91)	2023 (86.16)	2,208 (91.58)	41.043	**<0.001**	1,422 (93.86)	1,383 (90.93)	1,426 (82.76)	111.868	**<0.001**	3,480 (88.87)	751 (89.09)	9.073	**0.011**	1,404 (83.27)	2,827 (91.99)	84.034	**<0.001**	233 (87.59)	3,998 (88.98)	1.171	0.557

### Dangerous behaviors for drowning

In the past year, regarding dangerous drowning-related behaviors, 4.29% (204/4759) of adolescents reported having engaged in “water activities at unsupervised locations with no lifeguard/equipment,” while 10.74% (511/4759) reported “water activities accompanied by a guardian but with no lifeguard/equipment” (Table 3). In terms of gender, the prevalence of both behaviors was higher among males than females (*p* < 0.001). The incidence of these two risky behaviors varied significantly across grade levels (*p* < 0.001), moreover, with increasing grade level, the incidence of the two types of risky behaviors exhibited opposite trends. Regarding left-behind children, the incidence of the two types of risky behaviors did not differ significantly. Adolescents who answered all questions on drowning prevention correctly were defined as having mastered drowning prevention knowledge. The incidence of the two types of drowning-related risky behaviors was lower among adolescents with mastered drowning prevention knowledge compared to those without (*p* < 0.001).

**Table 3:** Comparison of dangerous behaviors for drowning in the past year across different groups, *n* (%).

Dangerous behaviors for drowning	n	Gender				Grade				Household registration				Boarding status			Left-behind children					Mastery of drowning prevention knowledge				
		Male	Female	*χ* ^2^	*P*	Primary school	Junior school	High school	*χ* ^2^	*P*	Local	Non-local	*χ* ^2^	*P*	Yes	No	*χ* ^2^	*P*	Yes	No	*χ* ^2^	*P*	Yes	No	*χ* ^2^	*P*
1. Water activities at unsupervised locations with no lifeguard/equipment	204 (4.29)	146 (6.22)	58 (2.41)	42.140	**<0.001**	58 (3.83)	65 (4.27)	81 (4.70)	1.497	0.473	163 (4.16)	41 (4.86)	0.831	0.362	81 (4.80)	123 (4.00)	1.705	0.203	13 (4.89)	191 (4.25)	0.248	0.639	106(3.54)	98 (5.56)	11.090	**0.001**
2. Water activities accompanied guardian with no lifeguard/equipment	511 (10.74)	310 (13.20)	201 (8.34)	29.386	**<0.001**	190 (12.54)	168 (11.05)	153 (8.88)	11.497	**0.003**	397 (10.14)	114 (13.52)	8.294	**0.004**	155 (9.19)	356 (11.58)	6.496	**0.011**	27 (10.15)	484 (10.77)	0.101	0.750	270 (9.01)	241(13.68)	25.234	**<0.001**

### Logistic regression analysis on dangerous behaviors for drowning

To ensure the stability of multivariate model and avoid overfitting, we only included variables with statistical significance (*P* < 0.05) from the univariate analysis in the multivariate model. As shown in Table 4, the multivariate regression analysis on whether water activities at unsupervised locations with no lifeguard/equipment was performed to identify major influences showed that gender (*P* < 0.001) and mastery of drowning prevention knowledge were positively associated (*p* = 0.006). Compared to females, males are more likely to engage in water activities at locations without lifeguards or safety equipment and without supervision from a caregiver (*OR* = 2.605, 95%*CI* = 1.909–3.554). Mastery of drowning prevention knowledge serves as a protective factor for such risky behaviors (*OR* = 0.671, 95%*CI* = 0.505–0.890).

**Table 4:** Multivariate logistic regression analysis for dangerous behavior 1 for drowning.

Variables	*Β*	SE	Wald	*P*	Exp(B) 95% CI
Male	0.957	0.159	36.462	<0.001	2.605 (1.909–3.554)
Mastery of drowning prevention knowledge	−0.399	0.144	7.640	0.006	0.671 (0.505–0.890)

As shown in Table 5, the multivariate regression analysis showed that males are more likely to engage in water activities at locations without lifeguards or safety equipment accompanied by the guardian (*OR* = 1.633, 95%*CI* = 1.352–1.971). Compared to primary school students, junior school students are more likely to engage in such behavior. Likewise, mastery of drowning prevention knowledge is a protective factor against such risky behaviors (*OR* = 0.586, 95%*CI* = 0.483–0.711).

**Table 5:** Multivariate logistic regression analysis for dangerous behavior 2 for drowning.

Variables	*Β*	SE	Wald	*P*	Exp(B) 95% CI
Male	0.490	0.096	25.872	<0.001	1.633 (1.352–1.971)
Grade			7.127	0.028	
Junior school	0.498	0.196	6.488	0.011	1.645 (1.122–2.414)
High school	0.271	0.176	2.375	0.123	1.311 (0.929–1.848)
Household registration	0.048	0.030	2.591	0.107	1.049 (0.725–1.439)
Mastery of drowning prevention knowledge	−0.534	0.098	29.509	<0.001	0.586 (0.483–0.711)

## Discussion

Drowning is a leading cause of accidental injury deaths, and the World Health Organization has identified it as a public health priority (19). As the world’s largest developing country, drowning remains a major cause of death among children and adolescents in China (20). The present study, drawing on a large and diverse sample of 4,759 Chinese children and adolescents, provides a comprehensive assessment of drowning prevention knowledge and related risk behaviors across different demographic groups. The survey results show that only 15.53% of respondents demonstrated the ability to swim continuously for 25 meters and tread water for 30 s. In most developed countries or regions, swimming is regarded as an essential life-saving skill and is promoted through school curricula or public initiatives. According to literature reports, up to 77.6% of children and adolescents in Sweden can swim, while the corresponding figures are approximately 50% in the United States and Hong Kong, China (21–23). A study by Brenner and colleagues found that formal swimming lessons reduced the risk of drowning among children between one and four years old by 88% (24). In China, swimming education and water safety awareness are still relatively underdeveloped, especially in rural areas, where access to swimming lessons and safe swimming environments is limited (25). This disparity highlights the importance of targeted drowning prevention programs tailored to specific demographic groups, such as those in rural regions or lower socio-economic statuses, where the risks of drowning may be higher due to lack of access to swimming facilities and insufficient water safety education.

The results of this study underline the significant gaps in drowning prevention awareness and risky behaviors among children and adolescents. Only the item “Don’t attempt to form a human chain to rescue a drowning person” had the lowest correct response rate, at 77.56%. And the second lowest was “Even skilled swimmers should never jump in immediately to save a drowning person,” at 88.91%. Despite the fact that the majority of the participants (90% or more) showed a basic understanding of drowning prevention principles such as not swimming alone or performing warm-up exercises before swimming, there were notable discrepancies in knowledge and behaviors across different demographic groups. These findings are crucial in understanding the areas that require focused intervention and can guide the development of more effective drowning prevention programs for children and adolescents.

One of the key observations from the study is the significant variation in drowning prevention knowledge across gender, household registration and boarding status. Males generally had lower knowledge rates compared to females, consistent with previous literature suggesting that boys tend to exhibit higher risk-taking behaviors and less awareness of water-related dangers (26–28). This gender gap highlights the need for targeted awareness campaigns aimed at young boys to address this disparity.

Age was another significant factor affecting drowning prevention knowledge. As expected, younger children in primary school demonstrated higher awareness than their older peers in junior and senior schools. This underscores the importance of continuous drowning prevention education as children age, ensuring that safety practices remain ingrained, even as they become more independent.

The impact of household registration and on drowning prevention knowledge is also noteworthy. Local students exhibited better knowledge compared to their non-local counterparts. This disparity could be linked to differences in access to resources, such as swimming lessons and safety programs, which are more readily available in urban areas. This finding emphasizes the need for more equitable distribution of drowning prevention resources, particularly in rural or less accessible areas, to ensure that all children, regardless of their background, receive the necessary education and support.

In addition, our findings indicate that boarding students demonstrated lower levels of drowning prevention knowledge compared to their non-boarding peers. This discrepancy may be related to reduced parental supervision (29), which are important sources of safety knowledge for children. Boarding students may also have fewer opportunities for informal discussions or reinforcement of safety practices outside of structured school activities. These factors suggest that interventions targeting boarding schools—such as regular water safety education sessions, supervised swimming training, and peer-led awareness programs—could help bridge this knowledge gap and enhance overall drowning prevention among this population.

Despite the absence of significant differences in drowning prevention knowledge and risky behaviors between left-behind children and other adolescents in this study, this may be due to the relatively small number of left-behind children in the survey. This group remains an important focus for targeted interventions, as left-behind children often face increased vulnerability due to reduced supervision and emotional support (30, 31). The study has shown that left-behind children in China are especially vulnerable to all types of unintentional injury because of poor care and supervision (32). They are at a higher risk of engaging in unsafe water behaviors, as demonstrated by the higher incidence of dangerous behaviors in this group. This absence of proper care and supervision is especially risky in rural areas, where a large number of children are left behind as their parents migrate for work in search of a better livelihood, and children are most often left in the care of family members, usually grandparents (33). While inadequate care and supervision for left-behind children is a concern in many regions, the issue is amplified in China by its sheer magnitude. Driven by extensive rural–urban migration, the country has an estimated 60 million left-behind children, accounting for 38% of its rural child population and 22% of all children. Notably, within this group, some 29 million reside without either parent, and over 2 million live in complete solitude (34). This indicates the need for targeted interventions that address both the emotional and educational needs of left-behind children, ensuring that they are not only aware of drowning risks but also equipped to make safer choices.

In terms of risky behaviors, our study reveals a concerning prevalence of unsafe water-related activities. A total of 4.29% of adolescents engaged in water activities at unsupervised locations without lifeguards, while 10.74% participated in water activities with a guardian but no lifeguard or safety equipment. The effect was especially evident among males, non-local students, boarding students, and those with poorer mastery of drowning prevention knowledge. The higher likelihood of engaging in dangerous water activities among these groups emphasizes the importance of practical interventions, such as improved supervision, community-based safety programs, and accessible life-saving skills training. The multivariate logistic regression analysis confirmed that gender, drowning prevention knowledge level were significant predictors of engaging in dangerous water-related behaviors. Males were more likely to engage in risky behaviors, as were older adolescents, especially those in junior school. These findings further support the need for targeted interventions addressing these high-risk groups, specifically target males, boarding students, and students with non-local household registration.

## Limitations

The cross-sectional study data were derived from self-administered questionnaires. Students surveyed need to recall information about themselves, which may lead to recall bias. Additionally, regarding certain sensitive questions, there was a possibility of exaggeration or concealment, resulting in socially desirable responses and causing reporting bias.

## Conclusion

This study highlights significant gaps in drowning prevention knowledge and engagement in risky water-related behaviors among children and adolescents in Ningbo, China. While overall awareness of basic drowning prevention principles is relatively high, certain groups—including males, boarding students, and students with non-local household registration—exhibit lower knowledge levels and higher participation in unsafe water activities. Despite the high levels of knowledge, the persistence of risky behaviors indicates that knowledge alone is insufficient to ensure safety. Factors such as peer influence, environmental conditions, inadequate supervision, and limited practical swimming skills likely contribute to ongoing unsafe practices. Although no significant differences were observed between left-behind children and their peers, this population still warrants attention due to potential vulnerabilities related to supervision and access to safety education. These findings underscore the need for targeted interventions that combine knowledge enhancement, practical safety education, and community-based supervision. Future programs should prioritize at-risk groups, promote safe swimming practices, and integrate family and school support to effectively reduce the incidence of drowning among children and adolescents.

## Data Availability

The datasets presented in this article are not readily available because the datasets and materials used and analyzed in the study are available from the author on reasonable request. Requests to access the datasets should be directed to zhzhong0220@163.com.
